# U-Plant and U-Plant DISCOVER: an open-source database for botanic garden collections management and public engagement

**DOI:** 10.1093/database/baag059

**Published:** 2026-09-21

**Authors:** Marco D’Antraccoli, Pietro Picconi, Giuseppe Maccioni, Davide Borgioli, Alessio Mo, Lorenzo Peruzzi

**Affiliations:** Botanic Garden and Museum, University Museum System, University of Pisa, via Luca Ghini 13, I-56126, Pisa, Italy; University IT Services, University of Pisa, Lungarno Pacinotti 43/44, I-56126, Pisa, Italy; University IT Services, University of Pisa, Lungarno Pacinotti 43/44, I-56126, Pisa, Italy; University IT Services, University of Pisa, Lungarno Pacinotti 43/44, I-56126, Pisa, Italy; Botanic Garden and Museum, University Museum System, University of Pisa, via Luca Ghini 13, I-56126, Pisa, Italy; Botanic Garden and Museum, University Museum System, University of Pisa, via Luca Ghini 13, I-56126, Pisa, Italy; PLANTSEED Lab, Department of Biology, University of Pisa, via Derna 1, I-56126, Pisa, Italy

## Abstract

Botanic gardens manage diverse plant collections and associated data that support research, conservation, education, and public engagement. Effective management of these collections requires robust, sustainable, and adaptable databases. Although several documentation solutions are available, they present different challenges: proprietary systems may involve costs, licensing constraints or limited institutional customization; locally developed databases may be difficult to maintain and update over time; spreadsheets may compromise data consistency and reliability. Here, we present U-Plant, a fully open-source database developed to support botanic garden collection management through a web-based interface. U-Plant records accession-level and individual-level data, including taxonomic identity, acquisition and source-locality information, garden location, condition history, and associated documentation. Its companion portal, U-Plant DISCOVER, provides public access to a curated subset of collection data through search tools, maps, and thematic visits, supporting both visitor engagement and external discovery of the collections. We describe the database structure, core functionalities, current implementation, and limitations, and outline priorities for future development, highlighting its potential as an open-source, community-supported database for botanic garden collections.

**GitHub repository link (U-Plant and U-Plant DISCOVER source code):**  https://github.com/Unipisa/U-Plant

**Database URL (U-Plant DISCOVER):**  https://uplantdiscover.sma.unipi.it/

## Introduction

Natural history collections constitute irreplaceable repositories of specimens and their associated data, offering unique opportunities to advance our understanding of biodiversity patterns and evolutionary processes that have shaped life on Earth [1]. They also provide a critical foundation for addressing the ongoing biodiversity loss observed in recent decades [2]. Among natural history collections, plant collections are particularly valuable for their role in supporting plant conservation from local to global scales, their broad utility for research, and their contribution to promoting botanical knowledge to the public [3,4]. These collections are typically housed in botanic gardens and arboreta (hereafter referred to as ‘botanic gardens’), which can be defined according to Botanic Garden Conservation International (BGCI, https://www.bgci.org/) as ‘*institutions holding documented collections of living plants for the purpose of scientific research, conservation, display, and education*’. According to this definition, building comprehensive and efficient databases for such collections is the cornerstone to support the mission and realise the full potential of these institutions [5].

There are currently more than 3000 botanic gardens worldwide, distributed in more than 180 countries [6,7]. Botanic gardens host more than 6 million accessions of living plants, representing ~80 000 taxa, roughly one-third of the currently known vascular plant species [8–10]. It is estimated that over 60 000 botanical experts, including plant scientists, educators, and specialist horticulturists are employed in these institutions [2], which are visited by over 300 million people annually worldwide [11]. This vast and highly interconnected network of collections, experts, users, and the public relies heavily on the effective management of specimens and their associated metadata, according to international modern best practices that encompass the accessioning of all specimens and the regular updating of databases. To support this strategic task, several software solutions are currently available, including proprietary systems such as BG-BASE (https://www.bg-base.com/), BRAHMS (https://herbaria.plants.ox.ac.uk/bol/), Hortis (https://www.hortis.com/), and IrisBG (https://irisbg.com/). Proprietary collection management solutions are often cost-prohibitive, prompting many botanic gardens to rely on independently developed databases or spreadsheets. However, these free alternatives have inherent limitations: custom databases may fail to evolve in response to changing needs due to budget constraints, and spreadsheet data are susceptible to accidental loss or overwriting. An open-source database for collection management can help address these problems while allowing the community of users to improve elements and functionalities in response to their needs.

Despite the availability of several proprietary solutions, no database specifically developed to meet the documentation standards of botanic garden collections has yet been released as a fully open-source and freely available tool. In recent years, however, new initiatives have started to emerge to address this gap. The Botalista project (https://botalista.community), initially developed by the Geneva Conservatory and Botanical Garden, is now maintained and further developed by a community of botanic gardens. It is a modular platform for managing diverse botanical data, ranging from living collections to seed banks and herbaria. Still evolving, it exemplifies the botanical community’s move towards open and standardized systems that improve data accessibility and interoperability. However, Botalista currently represents an intermediate model between proprietary and fully open-source solutions: a Community Edition for self-hosted installations has been announced but was not yet freely available online at the time of writing, whereas an Enterprise Edition is a licensed version reserved for association members that provides additional functionalities and includes partially closed-source components.

In this context, U-Plant builds upon this emerging movement by providing a fully open-source and publicly available collection database, with the source code of both the internal management system and the public-facing U-Plant DISCOVER portal released through a public repository. We present U-Plant as a free and functional database developed to support the management of botanic garden collections and to facilitate public access to curated collection data through U-Plant DISCOVER.

## Database construction and content

### Development principles

Implementing a well-structured database is a strategic task to ensure its effective use and its reliability over time. During the design phase, we followed three core principles:

1. **Cooperation and interoperability**: to design structures and workflows promoting collaboration and integration between curators, horticultural staff, and other personnel at the botanic garden.
2. **Clarity and simplicity**: to ensure a user-friendly interface that facilitates data entry and avoids redundancies or overcomplex fields, while retaining all essential information in line with international botanic garden standards.
3. **Bridging science and public engagement**: to develop the database as a tool capable of linking botany with public outreach [3], thereby supporting communication about plant biodiversity and conservation.

### General overview

The U-Plant project was initiated in 2018 and has been operationally adopted by the Botanic Garden and Museum of the University of Pisa (BGM-PI) since 2020. U-Plant aims to support accurate management and taxonomic standardization of botanic garden collections, facilitate role-based access for staff, and provide curated public access through the DISCOVER portal (https://uplantdiscover.sma.unipi.it/).

The U-Plant project was developed using an open-source approach, acknowledging that securing funding for its maintenance and development is challenging [12]. However, the emerging user community may play a key role in enhancing the tool by tailoring it to specific needs and identifying opportunities for improvement [13–15]. The source code of U-Plant and U-Plant DISCOVER is available in a public GitHub repository (https://github.com/Unipisa/U-Plant). The open-source release refers to the U-Plant and U-Plant DISCOVER source code, whereas the current BGM-PI deployment relies on a Microsoft-based server stack (see ‘Technical details’ section). Although U-Plant has been developed and is currently implemented at the BGM-PI, its architecture is intended to be adaptable to different institutional contexts. Adopting institutions can configure controlled vocabularies, organizational structures and operational workflows through administrator-managed Tables.

**Figure 1:**
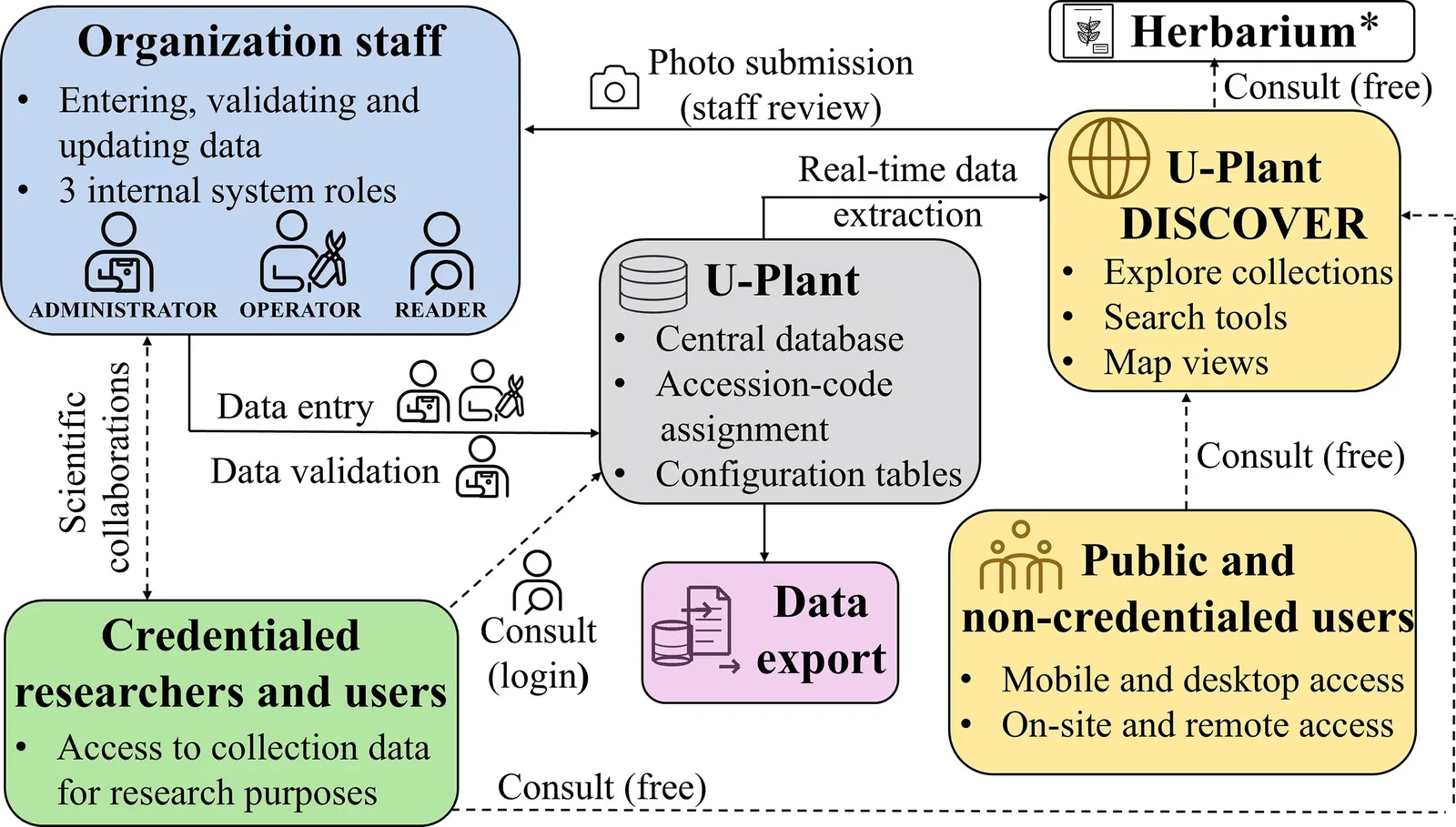
Schematic representation of the U-Plant workflow, showing the internal U-Plant database and the public-facing U-Plant DISCOVER portal and the main categories of users interacting with the system. Organization staff access U-Plant through three internal roles: administrators, operators, and readers. Administrators and operators enter and edit data through the web-based interface, whereas only administrators can validate accession records and manage configuration tables. Credentialed researchers and other authorized users may be granted login-based access to collection data for research purposes, while public users freely consult selected data through U-Plant DISCOVER. The portal retrieves in real time a selected subset of validated records from U-Plant and provides public tools for collection exploration, searches, and map views. The workflow also includes data export from U-Plant and public photo submission through U-Plant DISCOVER, with submitted images reviewed by staff. Solid arrows indicate data flows, whereas dashed arrows indicate data consultation or scientific collaboration links, as labelled. * In the BGM-PI implementation, U-Plant DISCOVER also links to the institution’s Virtual Herbarium for digitized herbarium specimens of the same species; this external resource is outside the scope of this work and is not included in the open-source release.

Within the internal U-Plant environment, each user account is associated with a user type, describing the user’s relationship with the botanic garden, and with one of three system roles: ‘Administrator’, ‘Operator’, or ‘Reader’ (Table 1, 1a–1c). System roles define access permissions within the database. Administrators manage the database’s core configuration data, supervise its content, manage the taxonomic backbone, and validate records entered by staff with ‘Operator’ credentials. Operators can enter and modify records within the database, except for those validated by administrators. Administrators may also grant selected operators delegated permissions to manage specific support tools (Table 1, section 5), while retaining control over core configuration Tables and record validation. Users with reader credentials have read-only access to all stored information. In contrast, the U-Plant DISCOVER portal is freely accessible to the public and provides a selected subset of metadata curated by the staff within U-Plant. A schematic overview of the database and its associated workflows is shown in Fig. 1.

**Table 1:** Structure of the U-Plant database, supported by practical examples from the Botanic Garden and Museum of Pisa (BGM-PI) to illustrate the meaning and intended use of each structure table.

	Tables	Description	Practical example from BGM-PI
**1**	***User management***		
1*a*	**Users**	A list of users, including their details and designated roles	–
1*b*	**User type**	Characterization of the user by specifying the relationship with the botanic garden	e.g. ‘Botanic Garden Staff’, ‘External Researcher’, and ‘Collaborator’
1*c*	System roles	Types of existing roles. The system currently recognizes three roles: ‘Administrator’, ‘Operator’, and ‘Reader’. Selected operators may be granted additional delegated permissions by administrators to manage specific support tools (see section 5 of this table)	–
1*d*	User-role link	Link between the users and system roles tables	–
	
**2**	***Accession tables***		
2*a*	Accession record	Table storing accession data	–
2*b*	**Acquisition mode**	How the material was obtained by the botanic garden	e.g. ‘Purchase’, ‘Deriving from research activities’, ‘Donation’, ‘Index Seminum’, and ‘Collected in the wild’
2*c*	**Material type**	Which type of plant material was acquired. The list is administrator-defined and may also include specialized material types such as pollen, spores, extracted DNA, or tissue samples where relevant	e.g. ‘Bare root’, ‘Bulb’, ‘Soil clump’, ‘Pot’, ‘Rhizome’, ‘Spore’, ‘Seed’, ‘Whole Plant’, and ‘Cutting’
2*d*	Country	List of the countries of the world	–
2*d*’	Region	List of the administrative regions of the country where the botanic garden is located	–
2*d*’’	Province	List of the administrative provinces of the country where the botanic garden is located	–
2*e*	**Source type**	Origin category of the material, indicating whether it is wild-sourced, cultivated, propagated, or of unknown origin	e.g. ‘Cultivated’, ‘Cultivated (from wild origin)’, ‘Propagated’, ‘Unknown’, and ‘Wild’
2*f*	**Arrival condition**	Qualitative condition of the material when it enters the botanic garden	e.g. a series of ordinal categories arranged in a qualitatively descending order, from ‘Optimal’ to ‘Poor’
2*g*	**Taxonomic uncertainty**	Degree of reliability of the identification of the material	e.g. ‘Confirmed Identification’, ‘Uncertain Identification’, ‘Pending Identification’, and ‘Verify subspecies’
2*h*	**Donor/Supplier**	Who provided the material and associated data	–
2*i*	**Collector**	Who collected the material (in case of wild sampling) and associated data	–
2*j*	**Verifier**	Who identified the material and associated data	–
2*k*	**Verifier type**	Type of verifier who identified or confirmed the material	e.g. ‘Internal staff’, ‘Specialist’, and ‘Amateur’
2*l*	**Documents**	Table storing metadata associated with uploaded documents, such as the ‘File Name’, ‘Format’, ‘Size', ‘Description’ (a text field to describe the document), ‘Entry Date’, and the user who uploaded the file	–
**3**	***Individual tables***		
3*a*	Individual record	Table storing individual-level data, including creation date and links to location, status, condition, images, and documents	–
3*b*	**Propagation method**	Method by which the individual was created or derived from the accession, including generative propagation, e.g. seed-derived, and vegetative propagation, e.g. clonal material	e.g. ‘Cutting’, ‘Division’, ‘Grafting’, ‘Potted’, ‘Seed packet created’, ‘Sown’, and ‘Field transplanted’
3*c*	**Sex**	Sex of the individual where relevant for the taxon or management context	e.g. ‘Hermaphrodite’ (H), ‘Female’ (F), and ‘Male’ (M)
3*d*	**Sector**	Physical areas of the botanic garden or associated staff facilities where individuals are located or stored	e.g. ‘Botanical School’, ‘Cedar Garden’, ‘Del Gratta Garden’, ‘Greenhouses’, ‘Myrtle Garden’, ‘Nursery’, and ‘Seed bank’
3*e*	**Collection**	Curatorial or thematic unit to which an individual is assigned. A collection may be defined according to taxonomic, geographical, conservation, educational, display or management criteria, and may include individuals located in different sectors	–
3*f*	**Label status**	The status of the label directly related to the individual	e.g. ‘Present’, ‘Ready for placement’, ‘Needs repair’, ‘To be modified’, and ‘In preparation’
3*f’*	**Label type**	The type of the label directly related to the individual	e.g. ‘Standard’, ‘Small’, and ‘Slat’
3*g*	**Individual status**	Management or life-cycle status of the individual within the collection	e.g. ‘Alive’, ‘Available’, ‘Dead’, ‘Out of stock’, ‘Mixed’, ‘Diseased’, ‘Donated’, and ‘Uncertain’
3*h*	**Condition**	An ordinal scale category used to assess the condition of the individual	e.g. ‘Optimal’, ‘Good’, ‘Average’, and ‘Poor’
3*i*	Individual record history	Table linking the given date (‘Entry date’) with other information, such as the ‘User’ (the person who added the historical record), the ‘Individual Status’ (see 3g), the ‘Condition’ (see 3h), and ‘Horticultural Operations’ (text field used to detail activities carried out on the individual)	–
3*j*	Images	Table storing metadata associated with uploaded images, such as the ‘Entry Date’, ‘File Name’, ‘Description’ (a text field to describe the image), a visibility flag (yes/no) to determine whether the image should be shown on U-Plant DISCOVER, the uploader’s name, and any applicable ‘Credits’.	–
3*k*	Individual/‘Search by themes’ link	A relationship between tables associating a specific individual with a proposed thematic visit (see Support Tools, ‘Search by themes’)	–
3*l*	**Documents**	Table storing metadata associated with uploaded documents, such as the ‘File Name’, ‘Format’, ‘Size', ‘Description’ (a text field to describe the document), ‘Entry Date’, and the user who uploaded the file	–
			
**4**	***Taxonomic backbone***		
4*a*	**Species**	Table storing species and all their ancillary data	–
4*b*	**Genus**	List of genera and their corresponding families	–
4*c*	**Family**	List of families	–
4*d*	**Biogeographical regionalization**	Broad biogeographical category used to characterize the native distribution of the taxon. For vascular plants, this may refer to floristic kingdoms or regions; for other organisms potentially included in botanic garden collections, such as fungi, the field can accommodate the most appropriate biogeographical or distributional classification.	e.g. floristic kingdoms, biogeographical regions or other distributional classifications adopted by the institution
4*e*	**Range**	The geographical area where the taxon occurs natively	–
4*f*	**CITES**	Categorization of species protection level according to the ‘Convention on International Trade in Endangered Species of Wild Fauna and Flora (CITES)’ (https://checklist.cites.org/)	–
4*g*	**IUCN**	Categorization of threatened species according to ‘International Union for Conservation of Nature (IUCN)’ Red List (https://www.iucnredlist.org/)	–
4*h*	**Taxonomic validation**	Categorization of taxonomic validation statuses for species	e.g. ‘WFO’ (WFO aligned), ‘N.D.’ (Pending Validation), and ‘A.A.’ (Manually Validated)
	
**5**	***Support tools***	Support tools are optional functional modules connected to the core database structure and may be expanded in future releases through additional add-on modules	
5*a*	**U-Plant DISCOVER ‘Search by themes’**	This table stores all data pertaining to the proposed thematic visits of the Botanic Garden. It includes the fields ‘Title’ and ‘Description’ (both textual), ‘Author’, ‘Creation Date’, and a binary ‘Active’ flag (yes/no) indicating whether the theme should be displayed on the U-Plant DISCOVER portal	BGM-PI offers permanent thematic visits such as ‘The Palms of the Botanic Garden’, ‘Monumental Trees’, and ‘Native Flora of Italy’, as well as seasonal proposals presented to the public during specific times of the year (e.g. ‘Blooms of the Winter Season’). See https://uplantdiscover.sma.unipi.it/Percorsi

**Figure 2:**
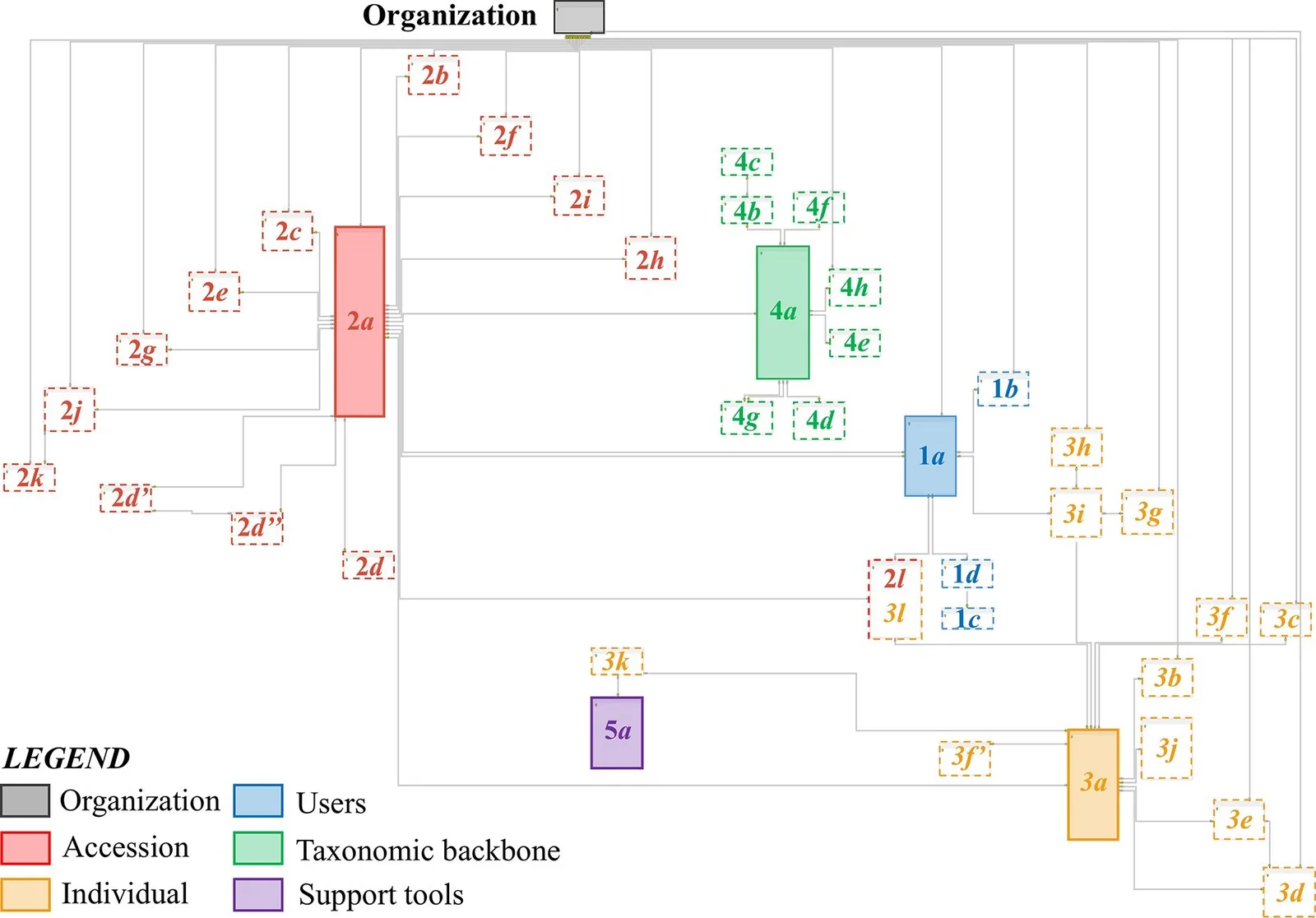
Structure of the U-Plant database. The ‘Organization’ entity is the top-level container for the database components associated with each adopting institution. The remaining table groups correspond to ‘Users’, ‘Accession’, ‘Individual’, ‘Taxonomic backbone’, and ‘Support tools’. Solid, filled boxes are the core nodes of the database architecture, whereas dashed boxes indicate related tables connected to these nodes. The relative size of the core boxes reflects the number of fields associated with the corresponding table. Codes (e.g. 3*a*, 3*b*) refer to the corresponding items in Table 1.

Table names in bold denote those that are to be populated by the Administrator through the ‘Administrator Tables’ panel. The top-level ‘Organization’ entity shown in Fig. 2 is not listed as a separate numbered table here, as it functions as the overarching institutional container within which user management, accession, individual, taxonomic backbone, and support-tool tables are organized.

## Technical details

The U-Plant database was developed using .NET and Microsoft SQL Server for data management, with minimal hardware requirements to facilitate deployment in diverse institutional settings. It can be hosted on a Windows Server configured with: (a) IIS (Internet Information Services) as a web server for serving pages via HTTPS, (b) .NET Core 8 Runtime to execute server-side pages, and (c) an SSL certificate to ensure secure site access. The database architecture supports efficient storage, retrieval, and update of records while ensuring data integrity and consistency. Records may include text files and images, with storage requirements proportional to the number of files. Georeferencing of records is supported through OpenStreetMap libraries, enabling mapping and spatial visualization. Additionally, the database offers optional integration with the DYMO LabelWriter™ 450, which depends on the DYMO Connect client-side service for printing code-strips.

## U-Plant database structure

The database comprises 40 Tables and 7 views, including 2 search-oriented views and 5 views dedicated to statistical summaries. Its architecture (Fig. 2) is structured around the ‘Organization’ entity, defined here as the institution adopting U-Plant. In the current release, each organization functions as an isolated data compartment: collection records cannot be viewed or exchanged across organizational boundaries. The organization Table is linked to the user-management Tables (Table 1, 1*a*–1*d*), which define user accounts, user types, system roles, and role assignments within each organization. Within each organization, U-Plant is built around two collection units: accessions (Fig. 2, 2*a*) and individuals (Fig. 2, 3*a*). The accession record is a distinct entry of plant material obtained from a given source at a specific time and source locality. Each accession receives a unique code in the format YYYY-NNNN, combining the year of accession with a sequential number. The individual record represents a managed unit derived from an accession and associated with a defined garden location, storage location or destination. Individuals derived from the same accession receive sequential suffixes appended to the accession code, e.g. YYYY-NNNN/0001, YYYY-NNNN/0002, and YYYY-NNNN/0003.

Taxonomic information is linked to accession records through dedicated backbone Tables for species, genus, and family, together with ancillary Tables for biogeographic regionalization, range, conservation status, and taxonomic validation status (Table 1, 4*a*–4*h*), while additional taxon-related information, such as common names, is stored in free-text fields. At initial setup, the taxonomic backbone can be populated through a bulk import from World Flora Online (WFO). Taxonomic validation is managed through administrator-controlled workflows that can query WFO in real time, compare stored names with WFO data and record the resulting validation status. This approach improves taxonomic consistency and enables periodic batch reassessment of nomenclatural status through the dedicated audit function. At the same time, WFO-based suggestions can be reviewed and, where necessary, locally accepted or modified by administrators. The taxonomic validation status (Table 1, 4*h*) records whether a name is WFO-aligned, pending validation or manually validated, allowing local curatorial treatments that differ from WFO to be explicitly documented.

**Figure 3:**
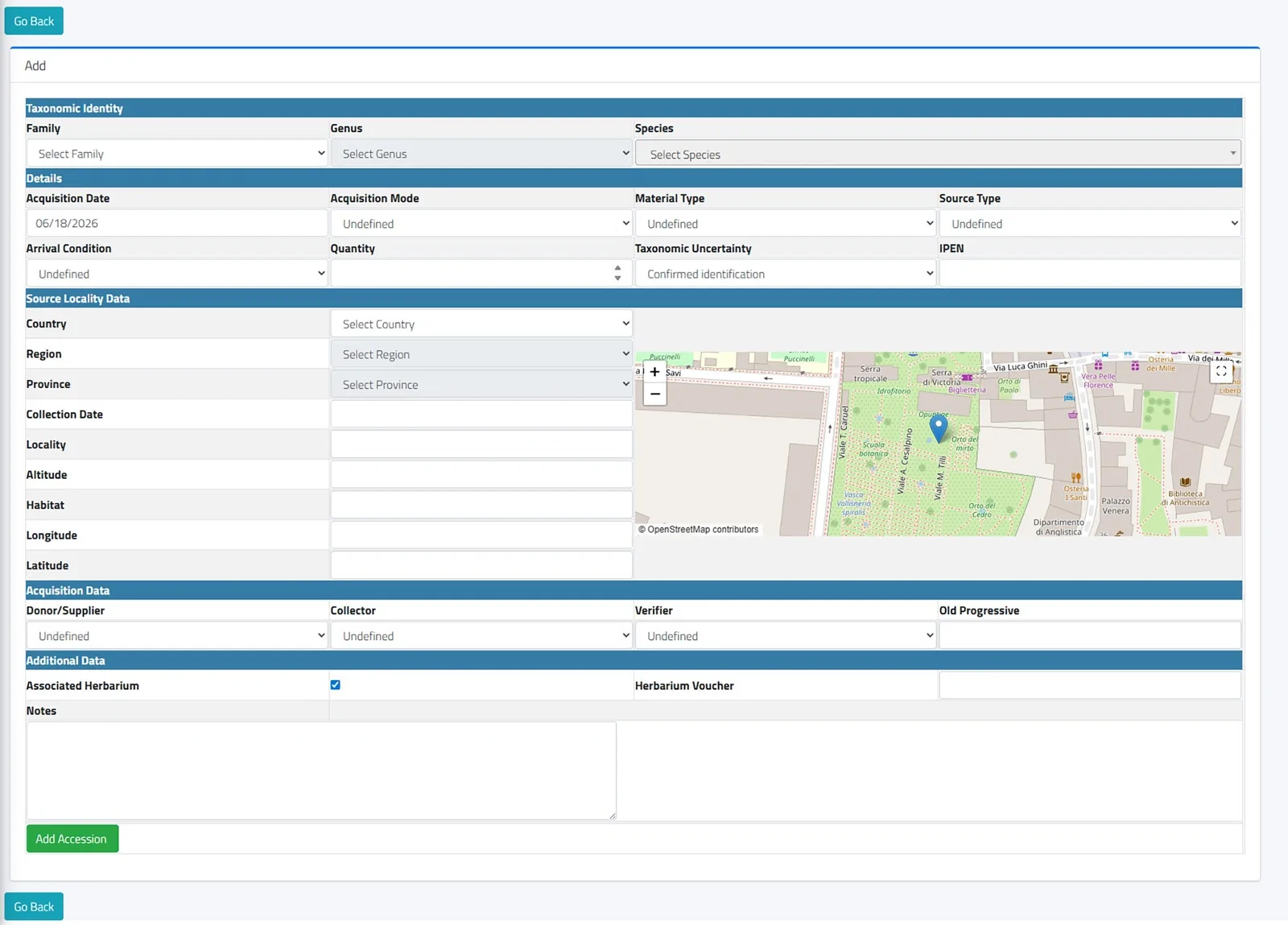
Accession record entry interface in U-Plant. The form used to create a new accession record is organized into thematic sections for taxonomic identity, accession details, source locality data, acquisition data, and additional data. Several fields are populated through controlled drop-down menus linked to administrator-managed configuration tables, and source locality data are supported by an integrated map for recording geographic information. The IPEN field can be completed manually by the user; if left blank, U-Plant automatically generates an IPEN code from the accession data recorded in the form. The example shows the ‘Associated Herbarium’ checkbox selected, with the corresponding field for entering the herbarium voucher associated with the accession displayed.

**Figure 4:**
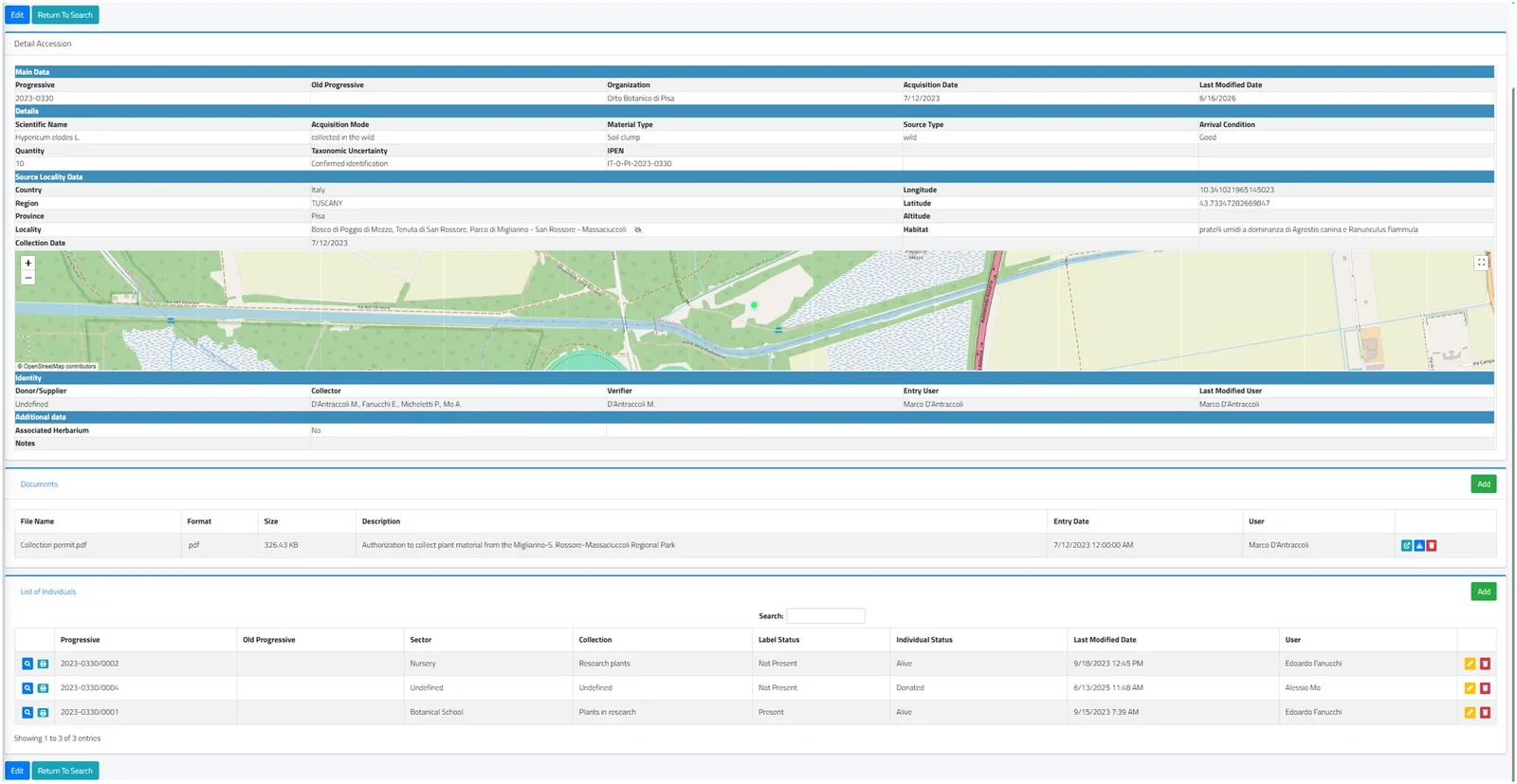
Accession record view in U-Plant, showing accession 2023-0330. The interface summarizes accession-level information, including scientific name and identification data, acquisition mode, material type, source type and source locality data, and associated geographic coordinates, when relevant. The integrated map displays the recorded source locality. The eye icon next to the locality field provides a visibility control for managing whether precise source-locality information is displayed publicly in U-Plant DISCOVER. The lower part of the page includes accession-level document storage, such as collection permits or other supporting files, and lists the individual records associated with the accession.

Accession records include taxonomic identity, managed through the taxonomic-backbone Tables (at least one taxonomic rank is mandatory in the record), acquisition mode (Table 1, 2*b*), material type (2*c*), source type (2*e*), arrival condition (2*f*), and IPEN code [16], which can be entered manually or automatically generated from accession data when the field is left blank, source locality data (2*d*–2*d*’’) complemented by a georeferenced map, donor/supplier (2*h*), collector (2*i*), verifier (2*j*–2*k*), legacy accession codes, an ‘Associated Herbarium’ flag with an optional herbarium voucher code field, and associated documents (2*l*), as shown in the accession entry form (Fig. 3) and in the corresponding archived record view (Fig. 4).

**Figure 5:**
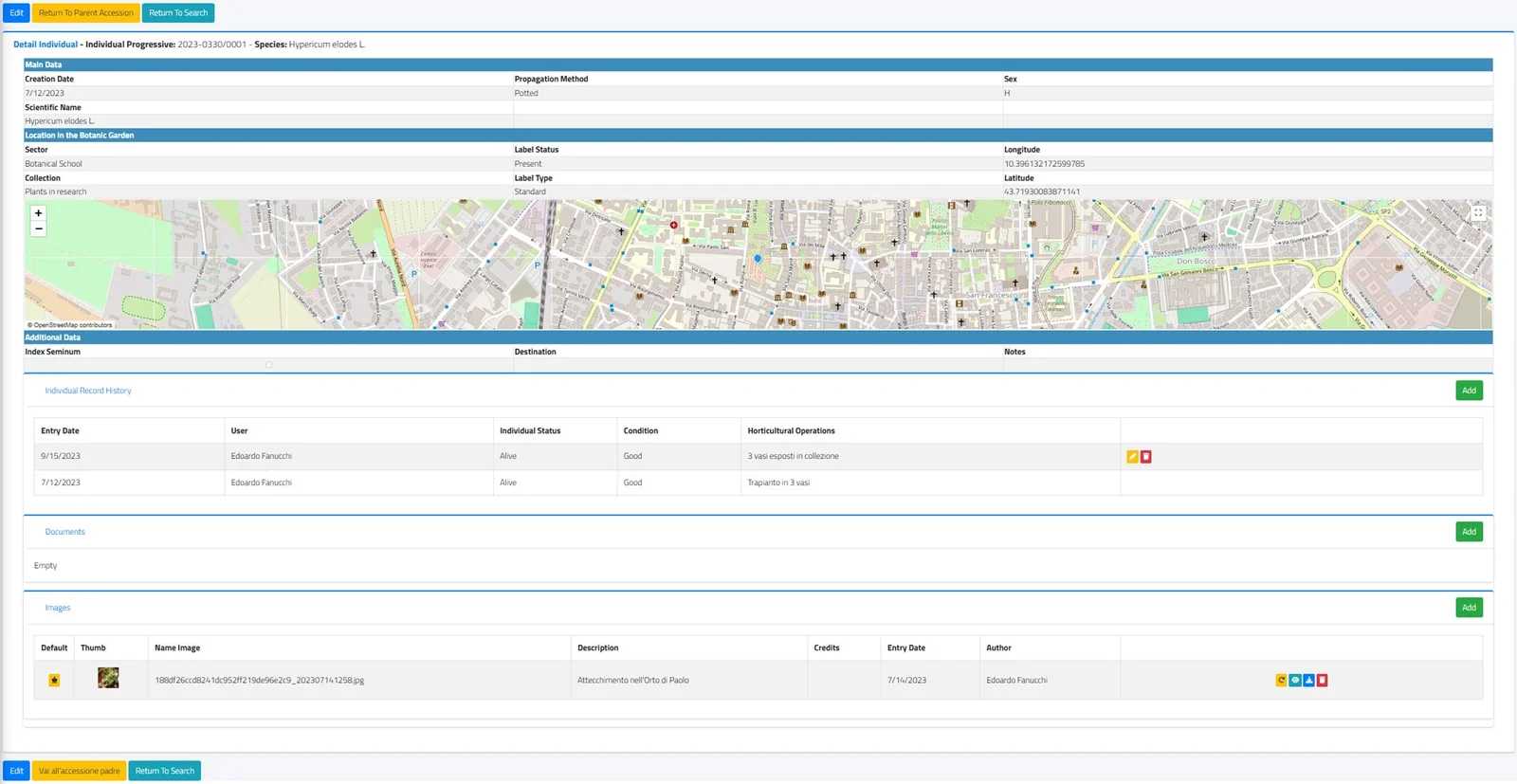
Individual record view in U-Plant, showing individual 2023-0330/0001. The upper section displays individual-level information, including main data, location within the botanic garden and the mapped position, label information and additional data. The middle section reports the individual record history, recording dated updates on status, condition and horticultural operations. The lower section includes areas for storing associated documents and photographic documentation, with image metadata and record-level actions shown where available.

Individual records (Fig. 5) store information on creation date, understood as the date on which the individual record is created, propagation method (Table 1, 3*b*) and sex where applicable (3*c*), sector and collection within the garden or associated facilities (3*d*–3*e*), label status and label type (3*f*–3*f*’), individual status and condition (3*g*–3*h*), dated record history (3*i*), images (3*j*), links to thematic visits in U-Plant DISCOVER (3*k*), and associated documents (3*l*). A free-text ‘Destination’ field further specifies the location, use or management context of the individual when this is not fully captured by sector and collection. The dated record history automatically stores the input date and user associated with each update, allowing changes in status, condition, and horticultural operations to be traced over time. Georeferencing at the accession level refers to the source locality of the plant material, whereas georeferencing at the individual level refers to the current mapped position within the botanic garden.

The combination of accession-level fields (e.g. ‘Material type’) and individual-level information (e.g. ‘Sector’, ‘Collection’, and ‘Destination’) allows U-Plant to record material beyond whole cultivated plants, including seeds, spores, extracted DNA, pollen, tissue samples, and other propagules. In the current release, these materials are managed within the general accession–individual schema; dedicated data models and domain-specific fields for seed banks, DNA banks or cryopreserved germplasm are not yet implemented. Both accession and individual records support the storage of associated documents (Table 1, 2*l* and 3*l*), including CITES documentation, phytosanitary certificates, plant passports, collecting permits, Material Transfer Agreements, and relevant scientific publications. Images associated with individual records are stored separately (Table 1, 3*j*), and their public visibility in U-Plant DISCOVER is controlled through a dedicated visibility flag.

**Figure 6:**
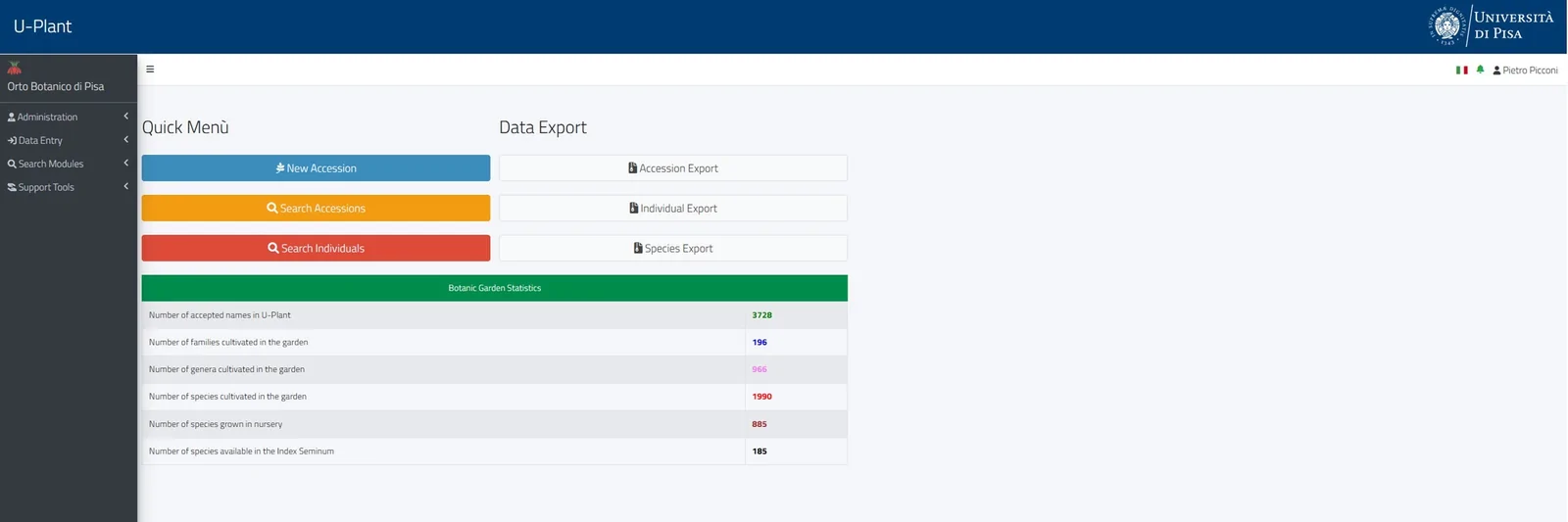
Homepage of the U-Plant database. The left-hand panel provides access to the main operational areas of the system, including administration, data-entry modules, search modules, and support tools. The central area includes a quick menu for creating new accession records and searching accession or individual records, together with data-export utilities for accessions, individuals, and species. The lower part of the page displays summary statistics for the collections managed by the organization. The appearance of the homepage varies according to user credentials; the view shown here corresponds to an administrator account.

The homepage of the U-Plant interface provides access to administrator-controlled configuration, record management, search modules, data export, and collection-level statistics (Fig. 6). Administrator credentials provide access to administration Tables, including user management, accession Tables, individual Tables, and the taxonomic backbone. Accession and individual forms are used to create and update records, whereas predefined search modules retrieve record-level summaries that can be exported as CSV files. The homepage provides access to these functions and separately displays aggregate statistics for the collections managed by each organization.

**Figure 7:**
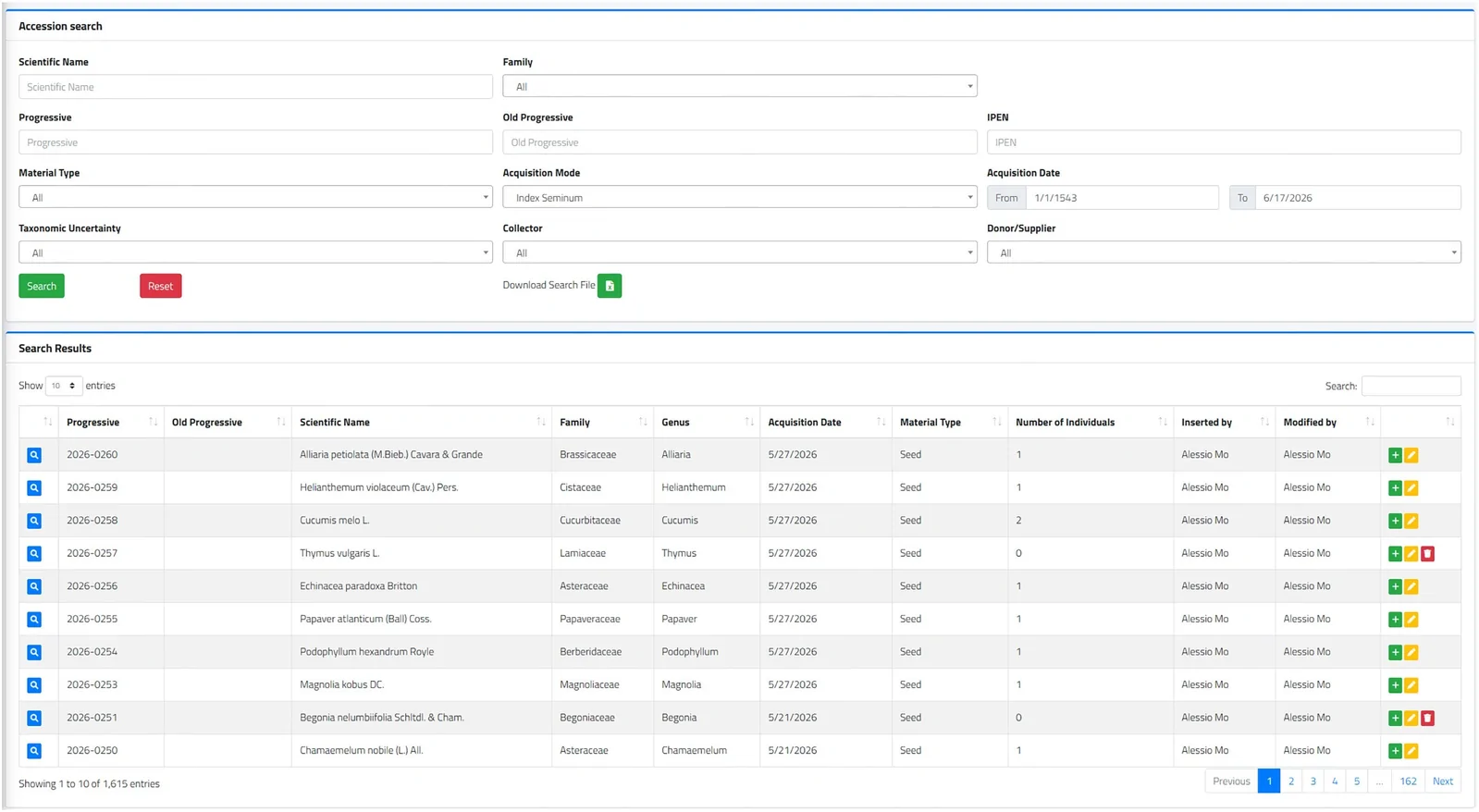
Accession search interface in U-Plant. The search form allows users to retrieve accession records through multiple filters, including scientific name, family, progressive number, old progressive number, IPEN code, material type, acquisition mode, acquisition date, taxonomic uncertainty, collector, and donor/supplier. Filtered results are displayed in a sortable and paginated table summarizing the main accession data, including progressive number, scientific name, family, genus, acquisition date, material type, number of associated individuals, and the users who inserted or last modified each record. The interface also allows users to download the filtered results as a CSV file and provides record-level action buttons, according to user permissions, for viewing or editing accession details and creating associated individual records.

The database includes three predefined search modules: ‘Recent Accessions’, ‘Search Accessions’, and ‘Search Individuals’. ‘Recent Accessions’ lists the 100 most recently entered accessions. ‘Search Accessions’ retrieves accession-level records and can be filtered by scientific name, family, accession code, legacy accession code, IPEN code, material type, acquisition mode, acquisition date range, taxonomic uncertainty, collector, and donor/supplier (Fig. 7). ‘Search Individuals’ retrieves individual-level records and can be filtered by scientific name, family, accession code, sector, collection, destination, individual creation date range, individual status, condition, and label status. Results from the accession and individual search modules can be downloaded as CSV files to support routine collection management, internal auditing, plant-exchange lists, and accession-level reporting.

## U-Plant DISCOVER

**Figure 8:**
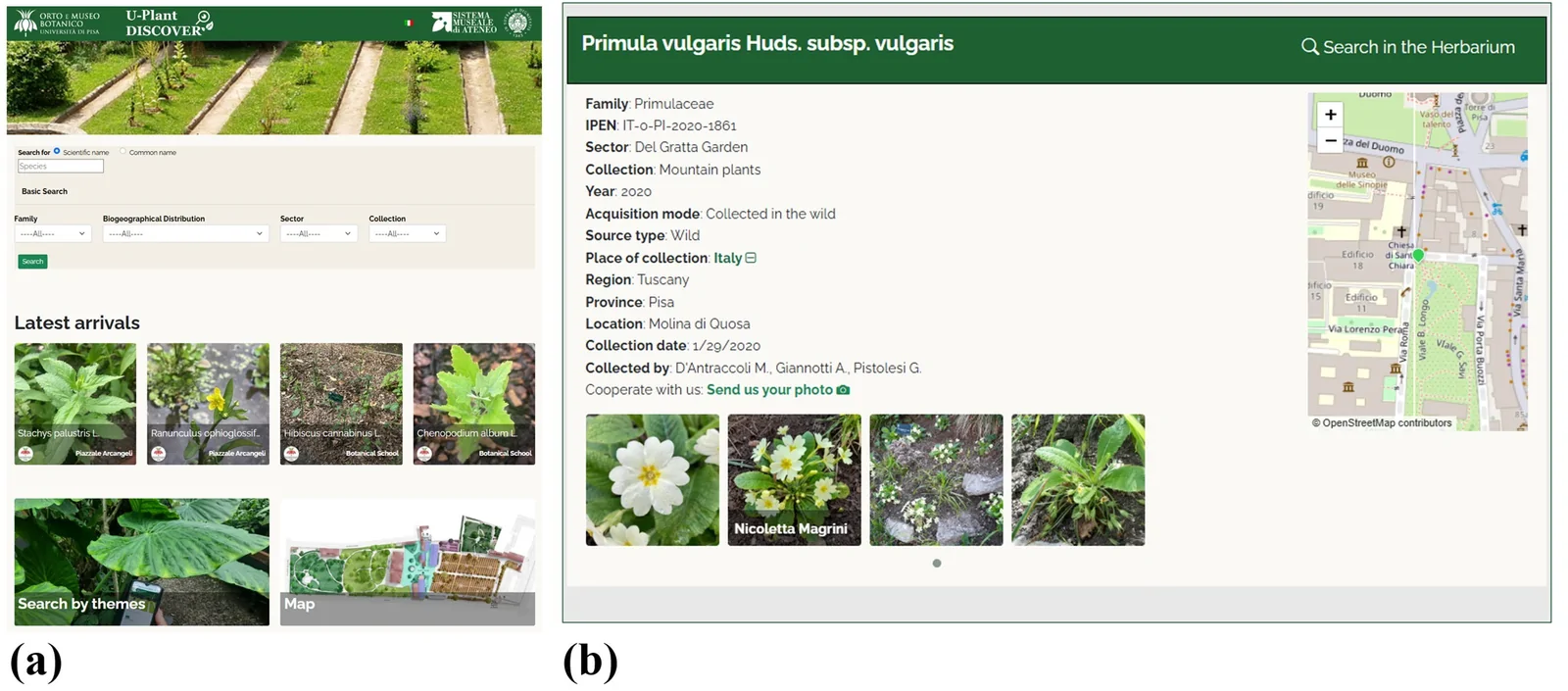
U-Plant DISCOVER public interface. (a) Homepage of U-Plant DISCOVER, freely accessible at https://uplantdiscover.sma.unipi.it/. The interface is organized into four main sections: basic and advanced search fields, the four most recently added individuals, thematic tours of the garden, and a static garden map supporting visitor navigation. (b) Full public record in U-Plant DISCOVER for an individual cultivated in the botanic garden. The page displays taxonomic and collection information, with the current garden position shown on the interactive map to the right. In the BGM-PI implementation, the record also provides a link to search the institution’s Virtual Herbarium. At the bottom of the page, a photo gallery is shown alongside a user engagement feature inviting visitors to contribute images of the individual through the ‘Send us your photo’ link.

U-Plant DISCOVER is a bilingual web interface that retrieves data from U-Plant in real time and displays only living individuals selected for public visibility. It is available in English and in the official language of the country in which the botanic garden is located. U-Plant DISCOVER is optimized for access via mobile devices such as smartphones. The homepage is organized into four main sections: search fields, a gallery showcasing the four most recently added individuals, thematic search options for exploring the plant collections, and a static map of the garden (Fig. 8a).

Searches can be performed either by scientific or common name, or through advanced queries based on criteria such as family, biogeographic regionalization, sector, or collection. Common names are stored as auxiliary metadata to support public-oriented searches and should therefore be interpreted as a user-support feature rather than as taxonomically authoritative data. Results are returned as a list of individuals matching the selected criteria, accompanied by a georeferenced map indicating their locations within the garden. Each individual entry contains a cover image and a summary of key information, along with a ‘Details’ button that opens the full record.

The full record (Fig. 8b) reports additional information such as taxonomic data, acquisition mode, source type, and source locality data (when available). For sensitive accessions, publicly displayed source-locality data can be restricted to country- or regional-level information rather than precise locations. The ‘Search in the Herbarium’ function is optional and institution-specific. At BGM-PI, it links U-Plant DISCOVER to the Virtual Herbarium of the University of Pisa (http://erbario.unipi.it/it), allowing users to search for herbarium specimens of the same species when available. This function requires an external searchable herbarium resource and is therefore not a core requirement for adopting U-Plant. At the bottom of each record, a photo gallery displays all archived images of the individual that are enabled for public visibility in U-Plant. Photographic documentation can also be enriched by users through the ‘Send us your photo’ feature, which currently relies on an external file-transfer service (WeTransfer, https://wetransfer.com/). This function is conceived as an optional user-engagement tool rather than as a core data-acquisition workflow: submitted images require manual review and curation by staff before they can be integrated into the database.

The ‘Search by themes’ section shows curated suggestions for thematic visits, each accompanied by a brief introduction, a list of relevant individuals, and their locations within the garden. Within this section, users can explore curated itineraries through the botanic garden that highlight specific topics or criteria of interest, thereby enhancing the interpretative and educational value of the collections. These thematic proposals may be permanently available to the public or temporarily activated according to seasonal topics (e.g. autumnal flowerings) or special events (e.g. commemorative occasions tied to a specific year or shorter time frame). While U-Plant DISCOVER is primarily designed to enhance the experience of general visitors, it is also fully accessible and useful to external botanic garden professionals and scientific experts, supporting research, plant material exchanges, and collaborative initiatives.

## Discussion and conclusions

U-Plant is a fully open-source database comprising an internal management component and the public-facing U-Plant DISCOVER portal, supporting collection management, museum and scientific work, and curated public access to collection data.

Compared with proprietary or hybrid models, which may involve costs, membership requirements, licensing constraints, or partially closed-source components, U-Plant offers a freely available and modifiable infrastructure that institutions can inspect, adopt, adapt, and further develop according to their own operational needs. In this sense, U-Plant may support greater institutional autonomy in collection-data management, especially where affordability, local configuration and control over future adaptations are priorities. In addition, while public dissemination may require optional or paid extensions in other systems, U-Plant integrates the internal management database and the public-facing U-Plant DISCOVER portal within the same open-source release. U-Plant therefore provides a working foundation for open collection management, with future extensions expected to emerge from structured collaboration among adopting institutions and technical contributors.

Botanic gardens are increasingly recognized as key players in global plant conservation, education, and biodiversity research [17–19]. In recent years, sharing data from living plant collections online has become increasingly common through platforms like BGCI’s PlantSearch [20], US National Plant Germplasm System [21] and, more broadly, Global Biodiversity Information Facility [22,23], where some botanic gardens have stored data about their collections. Although the trend towards increased data sharing is undoubtedly positive, unrestricted publication of full accession data may raise concerns for sensitive taxa, valuable collections, legally complex accessions, or records with restricted source-locality information [14]. These concerns often discourage collection curators from making their full datasets publicly available. In this respect, the separation between the internal U-Plant management environment and the public-facing U-Plant DISCOVER portal is particularly relevant, because it allows institutions to disseminate curated information while retaining control over sensitive accession-level data. In response to similar concerns, Holetschek and colleagues [14] initiated the ‘Gardens4Science’ project (http://gardens4science.biocase.org), which establishes a trusted network among botanic gardens, enabling unfiltered access to each member’s accession catalogue. Future implementations of U-Plant could enable data exchange across institutions through the top-level ‘Organization’ entity (see Fig. 2), thereby facilitating collaboration and plant material exchange among participating nodes.

Nevertheless, the development of open-source tools also poses challenges. Although U-Plant creates opportunities for further development through community contributions, its long-term expansion will depend on coordinated technical expertise, institutional commitment, shared development priorities, and a sustainable governance model. Further implementation in other botanic gardens will allow its flexibility to be assessed across collections of different sizes, scopes, and management practices. Community-based initiatives provide a useful reference, as they show how software development can be supported by institutional users, shared governance, coordinated technical roles, and collectively discussed development roadmaps. At present, U-Plant does not yet have a formal association, steering committee, or structured user board. Community feedback, bug reports, and implementation requests are currently collected through the public GitHub issue tracker, where users and developers can open issues and discuss proposed improvements. Future development should therefore move from this initial issue-based feedback mechanism towards a more structured governance framework involving collection managers, biodiversity-informatics specialists, software developers, and representatives of adopting institutions. Such a framework will be useful to prioritize implementation requests, coordinate releases, document database changes, maintain installation guidelines, develop import/export routines, and support integration with external taxonomic and biodiversity platforms. A further limitation concerns data migration: although U-Plant can import the WFO taxonomic backbone, legacy collection datasets still require manual entry or case-by-case conversion to the U-Plant data structure; robust import routines will therefore be important for broader adoption. In this context, U-Plant’s future development roadmap includes Darwin Core-aligned export capabilities (https://dwc.tdwg.org/), automated import routines for legacy datasets, enhanced query customization, analytical tools for monitoring collection composition and dynamics, dedicated modules for specialized material types, and tools for managing the *Index Seminum*, tree records, public display labels, and moderated image submissions through U-Plant DISCOVER.

Importantly, U-Plant DISCOVER contributes to bridging scientific data and public education. By allowing users to explore the collections through thematic visits, georeferenced maps, and interactive content, the system helps translate botanical knowledge into meaningful visitor experiences, supporting the educational mission of botanic gardens. Analytics for the period from 23 September 2024 to 23 September 2025 are provided in Supplementary Material 1. Current use is still limited relative to the overall number of garden visitors (ca. 2%), but these data provide a baseline for evaluating future outreach actions, including targeted communication campaigns, on-site QR codes, digital panels, and integration with visitor pathways.

Overall, U-Plant provides an open, functional, and extensible infrastructure for managing plant collections and sharing selected collection data with the public. Its value lies not only in its current functionalities, but also in its potential to support coordinated development around shared collection-management needs. By investing in open and well-governed digital infrastructures, botanic gardens can strengthen their capacity to manage collections, support research and conservation, and communicate botanical knowledge to wider audiences.

## Supplementary Material

baag059_Supplemental_File

## Data Availability

The source code of U-Plant and U-Plant DISCOVER is openly available in the GitHub repository at https://github.com/Unipisa/U-Plant. The public-facing U-Plant DISCOVER portal and the collection data made publicly available through it are accessible at https://uplantdiscover.sma.unipi.it/. Usage analytics reported in this article are provided in Supplementary Material 1.
