# Supplementary material for: U-Plant and U-Plant DISCOVER: an open-source database for botanic garden collections management and public engagement
Source: Database (Oxford). 2026 Sep 21;2026:baag059. doi: 10.1093/database/baag059 (PMC13591270; doi:10.1093/database/baag059)
Supplement: Supplementary file 1 [file baag059_supplemental_file.docx]

**SUPPLEMENTARY MATERIAL 1**

**Title:** U-Plant and U-Plant DISCOVER: an open-source database for botanic garden collections management and public engagement

**Authors:** Marco D’Antraccoli, Pietro Picconi, Giuseppe Maccioni, Davide Borgioli, Alessio Mo, Lorenzo Peruzzi

**Corresponding Author**: Marco D’Antraccoli ([marco.dantraccoli@unipi.it](mailto:marco.dantraccoli@unipi.it))

**Journal:** Database

**####### U-Plant DISCOVER annual analytics report #######**

(<https://uplantdiscover.sma.unipi.it/>)

**Analysis timeframe**: one year, from 23 September 2024 to 23 September 2025

**Source analytics**: Matomo ([www.matomo.org](http://www.matomo.org))

**Summary:**

1. General Overview
2. Audience Overview
3. Traffic Sources
4. Content Performance
5. Visitors Geography

**1. General Overview**

**Total visits**: 2,158

**Channel Types**:

- *Direct Entry*: 1,278 visits

- *Websites*: 471 visits (of which 96% originated from the official botanic garden website)

- *Search Engines*: 342 visits

- *Social Networks*: 67 visits

**Average visit duration**: 2 min 49 s

**Actions per visit** (e.g., page views, downloads, outlinks and internal site searches): 2.5

**Max actions in one visit**: 51

**Page views**: 4,637 (3,373 unique pageviews)

**Bounces**: 57% of visits bounced (i.e., left the website after viewing only one page)

**Outlinks**: 148 (138 unique)

The website U-Plant DISCOVER has been periodically promoted through the Facebook and Instagram pages of the Botanic Garden (e.g., [example 1](https://www.facebook.com/photo/?fbid=923557189788308&set=a.466462372164461), [example 2](https://www.facebook.com/photo/?fbid=524016873075677&set=a.466462372164461), [example 3](https://www.facebook.com/photo?fbid=610584514418912&set=a.466462372164461)). To date, no digital panels or stations providing access to the website have been installed in the Botanic Garden.

**2. Audience Overview**

**Visitors’ Statistics**

**New visitors**

- **New visits**: 1,314
- **Average visit duration for new visitors**: 1 min 55 s
- **Actions per new visit**: 2.1
- **New visits that bounced** (left the website after one page): 59%
- **Actions by the new visits**: 2,699

**Returning Visitors**

- **Returning visits**: 844
- **Average visit duration for returning visitors**: 4 min 15 s
- **Actions per returning visit**: 3.2
- **Returning visits that bounced** (left the website after one page): 55%
- **Actions by the returning visits**: 2,693

**Devices with available classification**: Smartphone (1,270), Desktop (861), Phablet (16), Tablet (2), Unknown (2)

**Website metrics over time**: the graphs below express the trends in six key website performance indicators (‘Actions’, ‘Bounces’, ‘New Visits’, ‘Returning Visits’, ‘Unique New Visitors’, and ‘Total Visits’). Each line represents the temporal variation in user engagement and visitor behaviour, highlighting fluctuations in overall website activity and audience dynamics.

**3. Traffic Sources**

The table below provides an overview of user engagement metrics across different acquisition channels: Direct Entry, Websites, Search Engines, and Social Networks. The data highlight differences in visitor behaviour and engagement depending on the traffic source, with Direct Entry exhibiting the highest overall activity.

| **Channel Type** | **Visits** | **Actions** | **Max actions in one visit** | **Average visit duration** | **Bounces** | **Unique visitors (daily sum)** |
| --- | --- | --- | --- | --- | --- | --- |
| Direct Entry | 1,278 | 2,988 | 51 | 2 min 52 s | 813 | 1,080 |
| Websites | 471 | 1,255 | 51 | 2 min 12 s | 206 | 440 |
| Search Engines | 342 | 1,042 | 29 | 3 min 58 s | 171 | 304 |
| Social Networks | 67 | 102 | 5 | 27 s | 50 | 67 |

The two graphs below show the daily trends in user visits.

**4. Content Performance**

**Five most visited Individual pages**: *Portulaca oleracea* L. (accession 2025-0451, 23 unique views, [link](https://uplantdiscover.sma.unipi.it/Home/Details?id=40091868-3bb6-44a0-ad3e-a9f0963dc6e2)), *Hedychium coronarium* J.Koenig (accession 2024-0524, 17 unique views, [link](https://uplantdiscover.sma.unipi.it/Home/Details?id=ea127f3ba08441c2bf3f7086b85eb05f)), *Santolina etrusca* (Lacaita) Marchi & D'Amato (accession 2020-2517, 15 unique views, [link](https://uplantdiscover.sma.unipi.it/Home/Details?id=183ad8d14f164feca0d2808248ce0023)), *Pinus nigra* J.F.Arnold subsp. *laricio* Palib. ex Maire (accession 2020-0020, 14 unique views, [link](https://uplantdiscover.sma.unipi.it/Home/Details?id=fa6a404a-ca3c-4889-9e1a-505d36e5d47e)), *Clematis flammula* L. (accession 2025-0376, 14 unique views, [link](https://uplantdiscover.sma.unipi.it/Home/Details?id=a01dbdbb-542b-456f-9b27-e2e5bdb4ee8a)).

**Number of visits to ‘Search by themes’**: 449 (335 unique views)

Most visited ‘Search by themes’ pages (Top 5)

- ‘Autumn at your feet’^*^ ([link](https://uplantdiscover.sma.unipi.it/Percorsi/Details?id=4aa6b7b3d7fd44a984d83541c8978175)): 185 (148 unique views)
- ‘Autumnal flowering’^*^ ([link](https://uplantdiscover.sma.unipi.it/Percorsi/Details?id=b327a26b1c2243f0922d78616f38e301)): 56 (46 unique views)
- ‘The palms of the Botanic Garden’ ([link](https://uplantdiscover.sma.unipi.it/Percorsi/Details?id=976d99bc-98ad-4961-bb9b-a38388b14897)): 46 (26 unique views)
- ‘Monumental trees' ([link](https://uplantdiscover.sma.unipi.it/Percorsi/Details?id=66535e3d-7f20-4eb7-9f9e-f3bb4d322851)): 40 (31 unique views)
- ‘Flora native to Italy’ ([link](https://uplantdiscover.sma.unipi.it/Percorsi/Details?id=00b4a2e8-d47e-446e-ae6a-e1b540bf0707)): 26 (20 unique views)

^*^ This thematic search on U-Plant DISCOVER was accessible to the public only from 21 September 2024 to 21 December 2024.

**Entry pages**

| **Entry page URL** | **Entrances** | **Bounces** | **Bounce rate** | **Average page load time** |
| --- | --- | --- | --- | --- |
| [Homepage](https://uplantdiscover.sma.unipi.it/Percorsi/Details?id=00b4a2e8-d47e-446e-ae6a-e1b540bf0707) | 1,741 | 955 | 55% | 9.44 s |
| [Search by themes](https://uplantdiscover.sma.unipi.it/Percorsi/) | 205 | 144 | 70% | 2.8 s |

**Exit pages**

| **Exit page URL** | **Exits** | **Unique page views** | **Exit rate** | **Average page load time** |
| --- | --- | --- | --- | --- |
| [Homepage](https://uplantdiscover.sma.unipi.it/Percorsi/Details?id=00b4a2e8-d47e-446e-ae6a-e1b540bf0707) | 1,350 | 1,781 | 76% | 9.44 s |
| [Search by themes](https://uplantdiscover.sma.unipi.it/Percorsi/) | 276 | 533 | 52% | 2.8 s |

**Top five external links visited:** [www.ortomuseobot.sma.unipi.it](http://www.ortomuseobot.sma.unipi.it) (56 clicks, 52 unique), erbario.unipi.it (40 clicks, 38 unique), [www.sma.unipi.it](http://www.sma.unipi.it) (19 clicks, 19 unique), [www.wetransfer.com](http://www.wetransfer.com) (19 clicks, 16 unique), [www.facebook.com](http://www.facebook.com) (5 clicks, 5 unique).

**Visits by visit number**

| **Visit number** | **Visits** | **% Visits** |
| --- | --- | --- |
| 1 visit | 1,314 | 61% |
| 2 visits | 288 | 13% |
| 3 visits | 93 | 4% |
| 4 visits | 48 | 2% |
| 5 visits | 37 | 2% |
| 6 visits | 29 | 1% |
| 7 visits | 25 | 1% |
| 8 visits | 22 | 1% |
| 9-14 visits | 94 | 4% |
| 15-25 visits | 94 | 4% |
| 26-50 visits | 99 | 5% |
| 51-100 visits | 15 | 1% |

**Visits per visit duration**

| **Time range** | **Visits** |
| --- | --- |
| 0–10s | 1,331 |
| 11–30s | 200 |
| 31–60s | 134 |
| 1-2 min | 120 |
| 2-4 min | 98 |
| 4-7 min | 73 |
| 7-10 min | 32 |
| 10-15 min | 41 |
| 15-30 min | 85 |
| 30+ min | 48 |

**Visits per number of pages**

| **N° of pages** | **Visits** |
| --- | --- |
| 1 page | 1,240 |
| 2 pages | 400 |
| 3 pages | 180 |
| 4 pages | 88 |
| 5 pages | 61 |
| 6-7 pages | 80 |
| 8-10 pages | 37 |
| 11-14 pages | 36 |
| 15-20 pages | 17 |
| 21+ pages | 19 |

**Visits by days since last visit**

| **Days since last visit** | **Visits** |
| --- | --- |
| New visits | 1,314 |
| 0 days | 456 |
| 1 day | 69 |
| 2 days | 34 |
| 3 days | 32 |
| 4 days | 28 |
| 5 days | 23 |
| 6 days | 18 |
| 7 days | 17 |
| 8-14 days | 59 |
| 15-30 days | 65 |
| 31-60 days | 24 |
| 61-120 days | 17 |
| 121-364 days | 2 |

**Incoming and outgoing traffic**: the Sankey diagram below illustrates the proportion of visits originating from internal pages, search engines, social networks, external websites, and direct entries. Outgoing traffic is represented by transitions to internal pages, downloads, external links, and exits. The central node summarizes the analysed pageviews (n = 2,451) and key engagement metrics, highlighting user navigation behaviour within the portal.

**5. Visitors Geography**

- **Continents:** Europe (2,033), North America (95), Asia (14), Oceania (6), South America (5), Africa (4), Central America (1).
- **Top 10 Countries:** Italy (1,493), United States (95), Germany (77), France (76), United Kingdom (74), Sweden (36), Netherlands (35), Poland (33), Albania (26), Ireland (25).
- **Top 10 Languages:** Italian (1,379), English (369), German (125), French (76), Dutch (47), Spanish (41), Polish (27), Swedish (13), Czech (12), Chinese (8).

**Visitor map. Parameter mapped: number of visits per country.**
